# Cleavage of Desmosomal Cadherins Promotes γ-Catenin Degradation and Benefits Wnt Signaling in Coxsackievirus B3-Induced Destruction of Cardiomyocytes

**DOI:** 10.3389/fmicb.2020.00767

**Published:** 2020-05-08

**Authors:** Guangze Zhao, Huifang M. Zhang, Ye Qiu, Xin Ye, Decheng Yang

**Affiliations:** ^1^Department of Pathology and Laboratory Medicine, University of British Columbia, Vancouver, BC, Canada; ^2^Centre for Heart Lung Innovation, St. Paul’s Hospital, Vancouver, BC, Canada; ^3^College of Biology, Hunan University, Changsha, China

**Keywords:** Coxsackievirus B3, desmocollin-2, desmoglein-2, γ-catenin, Wnt/β-catenin signal, intercalated disks, interferon-β

## Abstract

Coxsackievirus B3 (CVB3) is the primary etiologic agent of viral myocarditis, a major heart disease that occurs predominantly in children and young adolescents. In the heart, intercalated disks (ICD) are important structural formations that connect adjacent cardiomyocytes to maintain cardiac architecture and mediate signal communication. Deficiency in ICD components, such as desmosome proteins, leads to heart dysfunction. γ-catenin, a component protein of desmosomes, normally binds directly to desmocollin-2 and desmoglein-2. In this study, we found that CVB3 infection downregulated γ-catenin at the protein level but not the mRNA level in mouse HL-1 cardiomyocytes. We further found that this reduction of γ-catenin protein is a result of ubiquitin proteasome-mediated degradation, since the addition of proteasome inhibitor MG132 inhibited γ-catenin downregulation. In addition, we found that desmocollin-2 and desmoglein-2 were cleaved by both viral protease 3C and virus-activated cellular caspase, respectively. These cleavages led to the release of bound γ-catenin from the desmosome into the cytosol, resulting in rapid degradation of γ-catenin. Since γ-catenin shares high sequence homology with β-catenin in binding the TCF/LEF transcription factor, we further studied the effect of γ-catenin degradation on Wnt/β-catenin signaling. Luciferase assay showed that γ-catenin expression inhibited Wnt/β-catenin signaling. This finding was substantiated by qPCR to show that overexpression of γ-catenin downregulated transcription of Wnt signal target genes, c-myc and MMP9, while silencing γ-catenin upregulated these target genes. Finally, we demonstrated that γ-catenin expression inhibited CVB3 replication. In search for the underlying mechanism, we found that silencing γ-catenin caused down-regulation of interferon-β and its stimulated antiviral genes MDA5, MAVS, and ISG15. Taken together, our results indicate, for the first time, that CVB3 infection causes cardiomyocyte death through, at least in part, direct damage to the desmosome structure and reduction of γ-catenin protein, which in return promotes Wnt/β-catenin signaling and downregulates interferon-β stimulated immune responses.

## Introduction

Coxsackievirus B3 (CVB3) is a primary etiologic pathogen of viral myocarditis (Fairweather et al., 2012). Acute viral myocarditis can enter its chronic phase, dilated cardiomyopathy, which is a severe heart disease that may lead to heart failure or sudden death (Davies and McKenna, 1994; Mason, 2003). Numerous studies have shown that CVB3 replicates quickly in the heart after infection and causes direct cardiomyocyte injury or death, ultimately leading to loss of cardiac function (McManus et al., 1993). However, the pathway through which CVB3 infection causes direct cardiomyocyte destruction is still not fully understood.

Coxsackievirus B3 is a member of the *Picornaviridae* family. Its positive, single-stranded RNA genome can be directly translated into a polyprotein, which is cleaved into 11 mature viral proteins by viral proteases 2A and 3C. In addition to processing viral polyproteins to complete viral life cycle, proteases 2A and 3C also cleave a number of host proteins involved in host gene transcription and translation as well as signal transduction (Yang et al., 2003; Laitinen et al., 2016); these viral proteases thus play a critical role in viral pathogenesis.

Intercalated disks (ICD) are substantial structures that connect adjacent cardiomyocytes in the myocardium. The entire ICD structure is composed of three major complexes: desmosomes, gap junctions, and fascia adherens (Zhao et al., 2019). Desmosomes are essential for mechanically maintaining the architecture of cardiomyocytes (Sheikh et al., 2009), as well as for withstanding the strong forces imposed by heart contraction (Vermij et al., 2017). Disorganization of desmosome proteins in the myocardium results in multiple cardiac diseases, such as arrhythmogenic ventricle cardiomyopathy, which is caused by mutations in desmosome proteins (Campuzano et al., 2013; Rasmussen et al., 2014). In addition, several desmosome-related diseases, such as wooly hair and palmoplantar keratoderma have been reported (Rao et al., 1996; Carvajal-Huerta, 1998). These studies, however, largely focused on pharmacological reagent-induced disorganization of desmosomes in skin cells, and not on desmosome destruction in the heart due to viral infection, which has previously not been well investigated. A recent study from our laboratory found that reduction of ICD proteins in CVB3-infected cardiomyocytes is related to upregulation of certain microRNAs (Ye et al., 2014). For example, vinculin and α-catenin levels in the heart are decreased due to CVB3-induced upregulation of miR-21. We have also shown that γ-catenin (also called plakoglobin, encoded by the JUP gene), another important component of the desmosome, is robustly reduced during CVB3 infection; however, this decrease is not due to upregulation of miR-21, implying that other mechanisms are responsible for the downregulation of γ-catenin.

In addition to localizing in the desmosome as a structural protein, γ-catenin also participates in cell signaling in the cytosol. This protein is critical for desmosome assembly, especially the vertical linkage of desmosomal cadherins (desmoglein and desmocollin) to desmoplakin (Kowalczyk et al., 1999). Interestingly, in the presence of either desmoglein or desmocollin, γ-catenin’s half-life increases from 10–15 min to approximately 3–4 h (Kowalczyk et al., 1994), indicating that the binding of γ-catenin to neighboring desmosomal cadherins enhances its stability. γ-catenin is a close homolog of β-catenin, and they share many common interacting proteins, suggesting that γ-catenin may also be involved in the Wnt/β-catenin signaling pathway. However, the functional role of γ-catenin in Wnt/β-catenin signaling remains controversial and needs to be further investigated (Miravet et al., 2002; Maeda et al., 2004; Lombardi et al., 2011).

In this study, we first found that the reduction of γ-catenin was primarily due to the ubiquitin proteasome-mediated degradation caused by CVB3 infection. We further found that desmosome cadherins, desmocollin-2 (DSC-2) and desmoglein-2 (DSG-2), were cleaved by both CVB3 protease and virus-activated caspases. These cleavages promoted cellular relocalization and subsequent degradation of γ-catenin. To our knowledge, this is the first report on the cleavage of ICD proteins by cardiotropic viral proteases. In addition, we also found by luciferase assay that γ-catenin played an inhibitory role in Wnt/β-catenin signaling by competing with β-catenin to interact with the LEF/TCF transcription factor. As a result, overexpression of γ-catenin suppressed Wnt/β-catenin target gene transcription and also inhibited CVB3 replication. These data imply that CVB3-induced reduction of γ-catenin benefits CVB3 replication and generates a positive feedback effect on the destruction of ICD proteins and eventually cardiomyocyte death. Taken together, our studies revealed a previously unrecognized mechanism of CVB3-mediated cardiomyocyte destruction, based on reduction of cardiomyocyte structural integrity and inhibition of an integral signaling pathway.

## Materials and Methods

### Cell Culture, Animals, and Viral Infection

HeLa cells (ATCC) were cultured in Dulbecco’s modified eagle’s medium (DMEM) supplemented with 10% fetal bovine serum (FBS) (Sigma), 100 μg/ml penicillin-streptomycin and 2 mM glutamine (Thermo Fisher) at 37°C in 5% CO_2_. HL-1 cardiomyocytes, a mouse cardiac muscle cell line established from a cardiomyocyte tumor linage, were a gift from Dr. William C. Claycomb (Louisiana State University Health Science Center). These cells were cultured in Claycomb medium (Sigma) with 10% FBS, 100 μg/ml penicillin-streptomycin, 4 mM L-glutamine (Thermo Fisher) and 100 μM norepinephrine. For *in vivo* studies, 4-week-old male A/J mice were purchased from Jackson Laboratories (United States). Animal work was conducted by strictly following the Guide to the Care and Use of Experimental Animals – Canadian Council on Animal Care. All protocols were approved by the Animal Care Committee of Faculty of Medicine, University of British Columbia (protocol number A16-0093). The CVB3 (CG) strain was obtained from Dr. Charles Gauntt (University of Texas Health Science Center) and propagated in HeLa cells. Viral infection of cultured cells was conducted by incubation with CVB3 at a multiplicity of infection (MOI) of 10 for 1 h (for HeLa) or at a MOI of 50 for 2 h (for HL-1) in a serum free medium, washed with phosphate-buffered saline (PBS) (Thermo Fisher) twice, and then replenished with fresh medium containing 10% FBS. Mice were infected with CVB3 at a plaque forming unit (pfu) of 10^5^ or sham-infected with PBS by intraperitoneal injection. Infected mice were then euthanized at day 6 post-infection and the ventricular walls of the hearts were collected for analysis of target protein expression. The indicated MOI or pfu amounts and infection procedures were used throughout the entire work, unless otherwise specified.

### Cell Treatment With Inhibitor Against Proteasome, GSK3, or Caspase

HeLa cells were pre-treated with proteasome inhibitor MG132 (Selleckchem) to block protein degradation at 5 μM or an equal volume of DMSO for 1 h and then infected with CVB3. Cellular proteins were then collected at 5 and 7 h post infection (pi) to detect target protein degradation by Western blot analysis. To inhibit caspases, HeLa cells were pre-treated with z-VAD-fmk (Selleckchem) at 50 μM or equal volume of DMSO for 1 h and then infected with CVB3. Cellular proteins were collected at 5 and 7 h pi to detect the blockage of caspase-3-mediated cleavage of target protein by Western blot analysis. To inhibit glucose synthase kinase-3 (GSK3), HL-1 cells were pre-treated with inhibitor SB216763 (Selleckchem) at 40 μM or equal volume of DMSO for 1 h and then infected with CVB3. Cellular proteins were then collected at 12 and 24 h pi to determine the effect of inhibition on γ-catenin level by Western blot analysis. All these inhibitors were used at a lower concentration that could not significantly affect CVB3 replication based on our previous experience (Yuan et al., 2005b; Qiu et al., 2017; Zhang et al., 2020).

### Transfection of Plasmids and siRNAs

All the plasmids used in this study were purchased from Addgene (United States), including 1105-desmoglein2-FLAG-eGFP, 476-desmocollin2-myc and 330-plakoglobin-myc. The γ-catenin siRNA (human) and DSC-2 siRNA (human) were purchased from Santa Cruz (United States). To perform the transient transfection, HeLa cells (2 × 10^5^) were seeded in 6-well plates and grown to 60∼80% confluence (for plasmid) or 30∼40% confluence (for siRNA) before transfection. Plasmid DNA was then transfected into the cells using Lipofectamine 2000 (Life Technologies) and Opti-MEM^TM^ (Thermo Fisher) complex for 6 h, and then incubated for 48 h after replacing the medium with DMEM containing 10% FBS. The siRNA was transfected using the same procedures as those described for plasmid except that Oligofectamine was used as transfection reagent. Scrambled siRNA and empty vector were used as controls in these transfections, respectively. Further, CVB3 infection was conducted at 36 h (for plasmid) or 48 h (for siRNA) post transfection before final analysis.

### RNA Extraction and Quantitative Real-Time PCR (qPCR)

Total cellular RNAs were isolated using the PureLink^TM^ RNA mini kit (Invitrogen) according to the manufacturer’s instructions. cDNAs were then synthesized by reverse transcription using 5 μg of RNA and the SuperScript IV First-Strand Synthesis System (Invitrogen). The target genes were detected by qPCR using 2 × SYBR Green qPCR Master Mix (Bimake), 100 ng of first-strand cDNA and 1 μM of each primer. Glyceraldehyde 3-phosphate dehydrogenase (GAPDH) expression level was used as the endogenous control to normalize the data. All PCR experiments were performed in triplicates and repeated twice. The PCR primer sequences are listed in Supplementary Table S1.

### Western Blot Analysis

Cultured cells were washed with ice cold PBS twice and then lysed with an appropriate volume of RIPA Lysis and Extraction Buffer (Thermo Fisher) on ice for 20 min. Cell lysates were centrifuged at 13,000 × *g* for 20 min at 4°C and the protein-containing supernatant was collected. The protein concentration was determined by BCA Protein Assay (Thermo Fisher). For preparing mouse heart tissue lysates, the heart tissue sections were first rinsed with PBS to remove the blood and then lysed in RIPA lysis buffer using the TissueLyser LT (Qiagen). Samples were briefly sonicated at 50 Hz for 5 min to release the proteins. The protein-containing supernatants were collected by centrifugation and protein concentrations were determined as described above. Western blot was conducted by following the standard method (Qiu et al., 2017). Briefly, proteins were separated by sodium dodecyl sulfate-polyacrylamide gel electrophoresis (SDS-PAGE) with a corresponding polyacrylamide concentration according to the protein molecular weights and transferred onto BioTrace^TM^ NT nitrocellulose membranes. The membranes were blocked with 5% skim milk in Tris-buffered saline with 0.1% Tween 20 (TBST) for 1 h and then incubated overnight with one of the following primary antibodies: monoclonal rat anti-VP1 (Doka), polyclonal rabbit anti-γ-catenin (Cell Signaling), monoclonal mouse anti-desmocollin-2 and monoclonal rabbit anti-desmoglein-2 (Abcam), monoclonal mouse anti-cleaved-caspase-3 (Cell Signaling), monoclonal rabbit anti-c-myc (Abcam) and K48-linkage specific polyubiquitin rabbit antibody (Cell Signaling) and monoclonal mouse anti-β-actin-HRP (Santa Cruz). After three washes with TBST, each blot was further incubated with a corresponding secondary antibody (goat anti-mouse or donkey anti-rabbit IgG) conjugated to horseradish peroxidase (Amersham). Detection was carried out by enhanced chemiluminescence (Amersham) as per the manufacturer’s instructions. β-actin was used as the loading control. Signal intensities were quantified by densitometric analysis using the NIH ImageJ program and then normalized to the loading control.

### *In vitro* Cleavage Assay

The *in vitro* cleavage assay was conducted as described previously (Skern and Liebig, 1994). In brief, cultured HeLa cells were collected in RIPA lysis buffer and centrifuged at 13,000 × *g* at 4°C for 20 min. 10 μl of supernatant containing 25 μg of total protein were mixed with 20 μl of reaction buffer (20 mM PH 7.4 Hepes, 150 mM potassium acetate, and 1 mM DDT) and 10 μl of recombinant wild-type or mutant (inactive catalytic site) CVB3 protease 2A or 3C (kind gifts from Drs. Eric Jan and Honglin Luo, respectively) containing 10 μg of protease (Jagdeo et al., 2018). This reaction was then incubated at 37°C for defined periods of time (0 min, 15 min, 30 min, 1 h, and 2 h) and stopped by the addition of SDS-PAGE sample buffer. Cleavage products were detected by Western blot analysis as described above.

### Viral Plaque Assay

Viral titer was determined following the procedure published previously (Yuan et al., 2005a). Briefly, samples of cells and medium from plates receiving the various treatments were freeze-thawed and then centrifuged at 4,000 × *g* to collect the supernatant containing the viruses. HeLa cells were seeded onto 6-well plates (8 × 10^5^ cells/well) and incubated at 37°C for 20 h to reach confluence of approximately 90% and then washed with PBS and overlaid with 800 μl of virus-containing samples serially diluted (10^–^^1^ to 10^–^^7^) in cell culture medium. After a viral adsorption period of 60 min at 37°C, the supernatant was removed and the cells were overlaid with 2 ml of sterilized soft (0.75%) Bacto-agar-minimal essential medium, cultured at 37°C for 72 h, fixed with Carnoy’s fixative for 30 min, stained with 1% crystal violet for 2 min and then washed with PBS twice. The number of plaques was counted, and the virus titer was calculated as the pfu/ml. All the assays were conducted three times.

### Immunofluorescence Staining and Confocal Microscopy

Cells cultured in μ-Slide 8 Well ibiTreat:#1.5 polymer coverslip (Ibidi, Cat. No. 80826) were washed with PBS and fixed with 4% paraformaldehyde in PBS for 20 min at room temperature. After removing the fixative, cells were further washed with fresh 0.1 M glycine in PBS for three times (5 min each). Fixed cells were permeabilized with 0.1% Triton X-100 in PBS for 2 min, washed with PBS three times and then blocked with 3% bovine serum albumin (BSA) in PBS for 1 h at room temperature, which was followed by removing the blocking buffer and incubating the cells with a corresponding primary antibody (same antibodies as for the Western blot) at 4°C overnight. The cells were then washed with PBS and incubated with corresponding secondary antibodies: goat anti-rabbit IgG labeled with Alexa Fluor 488 (green), goat anti-mouse IgG labeled with Alexa Flour 594 (red), or goat anti-rat IgG labeled with Alexa Flour 633 (purple) (Invitrogen). The nuclei were stained with 4′,6′-diamidine-2′-phenylindole dihydrochloride (DAPI) (Sigma). Images were captured using a Zeiss LSM 880 confocal microscope and merged using the Zen Black and Zen Blue programs. To quantify the intensity of fluorescence signals, the same imaging setting parameters were applied to all the samples. Relative protein levels were then quantified by converting the captured photos to 8 bit-pictures and measuring the mean gray value of the entire image using the NIH ImageJ program.

### Immunoprecipitation and Ubiquitination Assay

Cell lysates of CVB3-infected and sham-infected cells were prepared using lysis buffer containing 50 mM Tris HCl, pH 7.4, 150 mM NaCl, 1 mM EDTA, 1% Triton X-100, and protease inhibitor cocktail (Roche), and centrifuged at 14,000 × *g* for 5 min at 4°C. After preclearing for 2 h, the supernatants were immunoprecipitated overnight using the anti-myc antibody and the Pierce Crosslink IP Kit (Thermo Fisher) according to the manufacturer’s instructions. The enriched proteins were separated by a 6% SDS-PAGE and then immunoblotted using the K48-linkage anti-polyubiquitin antibody (Cell Signaling).

### Dual Luciferase Assay

The β-catenin firefly reporter (M50 Super 8 × TOPFlash), the Renilla reporter (pcDNA-RLuc8) and 330-plakoglobin-myc plasmids were purchased from Addgene. The β-catenin firefly reporter plasmid contains a TCF/LEF binding motif upstream of a firefly luciferase reporter, while the Renilla reporter is a constitutively expressed reporter, serving as a control. Thus, the expression of firefly luciferase is regulated by the activity of TCF/LEF binding proteins (such as β-catenin). To determine the effect of γ-catenin on the TCF/LEF-regulated luciferase activity, HeLa cells were co-transfected with two luciferase reporters (M50 Super 8 × TOPFlash and pcDNA-RLuc8) and the 330-plakoglobin-myc plasmid. For the negative control group, an empty vector was used to replace the 330-plakoglobin-myc plasmid for co-transfection. At 36 h post transfection, cells were infected with CVB3 for 6 h and then the cell lysates were harvested for luciferase assay on a Tecan GENios luminescence reader to determine the relative luciferase activity (firefly/Renilla) using the Dual-Luciferase Reporter Assay System (Promega) following the manufacturer’s instructions. Each treatment was verified by three biological repeats.

### Statistical Analysis

Two-way ANOVA analysis of variances was performed for determining differences between groups on multiple variables, and Student’s *t-*test was conducted to analyze the paired groups. Results are shown as means ± standard deviation (SD) of three independent experiments. A *p-*value less than 0.05 (indicated by ^∗^) was considered statistically significant. Additionally, ^∗∗^*p* < 0.01; ^∗∗∗^*p* < 0.001; ^****^p < 0.0001.

## Results

### CVB3 Infection Decreases γ-Catenin Through Ubiquitin Proteasome-Mediated Degradation

**FIGURE 1:**
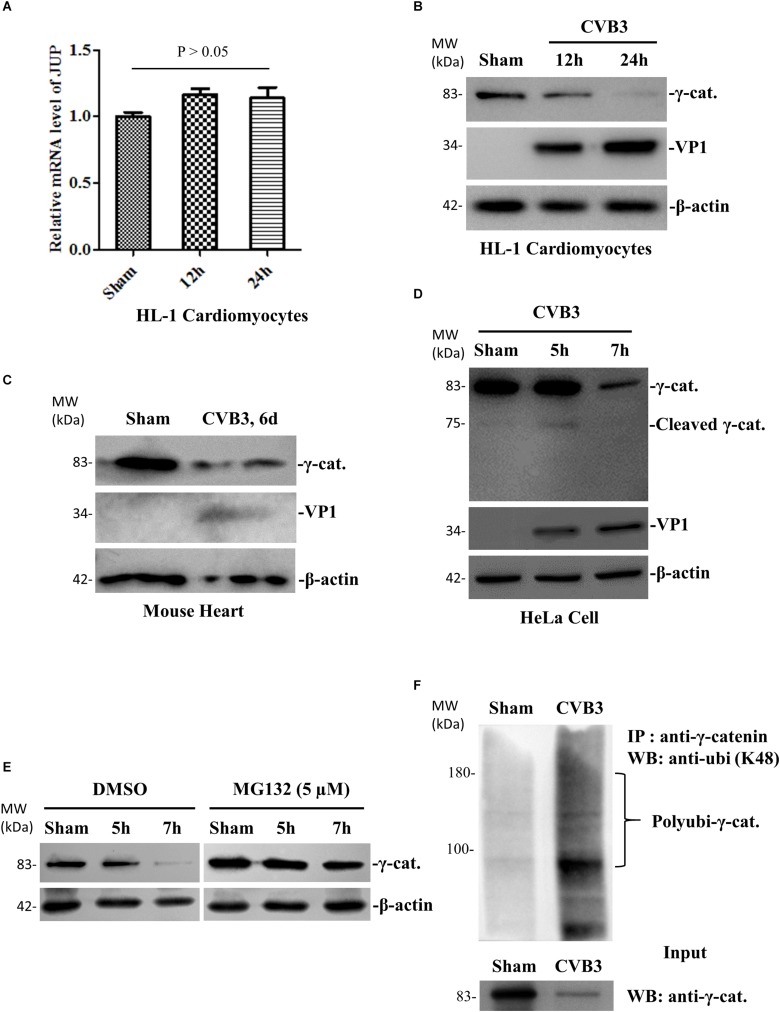
CVB3 infection decreases γ-catenin level through the ubiquitin-proteasome pathway of protein degradation. **(A)** HL-1 mouse cardiomyocytes were infected with CVB3 at a MOI of 50 or sham-infected with PBS and the cell lysates were harvested at the indicated time points pi. Total cellular RNAs were isolated for qPCR to measure the mRNA of γ-catenin (encoded by JUP gene). The relative level of the mRNA at each time point was normalized to the level of GAPDH in the sample. Three biological repeats were performed for each assay, and the Student’s *t*-test was used to analyze the data. *p* > 0.05 (not significant). **(B)** HL-1 mouse cardiomyocytes were infected with CVB3 or sham-infected as described in **(A)** and the cell lysates were harvested at the indicated time points pi for Western blot analysis of γ-catenin and CVB3 VP1 protein. β-actin was used as a loading control. **(C)** 4-week-old A/J mice were infected with CVB3 at 10^5^ pfu or sham-infected with PBS. At 6 days pi, the mice were sacrificed and the heart tissues were homogenized for Western blot analysis of γ-catenin protein. **(D)** HeLa cells were infected with CVB3 at a MOI of 10 or sham-infected with PBS and cell lysates were prepared for Western blot analysis of γ-catenin cleavage. **(E)** HeLa cells were pretreated with 5 μM MG132 and then infected with CVB3 at a MOI of 10. At the indicated time points pi, the cellular proteins were subjected to Western blot analysis of γ-catenin protein. **(F)** HeLa cells were infected or sham-infected as described above for 7 h. Cell lysates were prepared and first immunoprecipitated (IP) with the anti-γ-catenin antibody and then subjected to Western blot (WB) analysis of K48-linked polyubiquitin chains. The input (non-immunoprecipitated) samples were detected with the anti-γ-catenin antibody.

Our previous studies demonstrated downregulation of γ-catenin during CVB3 infection (Ye et al., 2014). To further investigate how γ-catenin levels are decreased, we first asked whether γ-catenin expression is downregulated at the transcriptional level. qPCR using total mouse HL-1 cardiomyocyte RNAs and specific primers targeting the γ-catenin gene (Supplementary Table S1) showed that CVB3 infection did not alter the mRNA levels of γ-catenin (encoded by the JUP gene) (Figure 1A). We then measured the changes in protein translation as well as potential post-translational cleavage and degradation. We first confirmed, by Western blot, that γ-catenin protein levels are downregulated in CVB3-infected HL-1 cardiomyocytes (Figure 1B) and in mouse hearts (Figure 1C). To determine if γ-catenin is cleaved by viral and/or cellular proteases, we performed a Western blot using a short running SDS-PAGE gel to capture the cleavage products. Although a very faint band (∼75 kDa) appeared at 5 h pi, a similar band also appeared in the sham-infected control, suggesting that this cleavage is not by CVB3 protease but rather by activated cellular protease (Figure 1D). Considering the faintness of this band at 5 h pi and its disappearance at 7 h pi, the reduction of γ-catenin might be mainly due to degradation. To verify this, we treated HeLa cells with proteasome inhibitor MG-132 for 1 h before CVB3 infection. In this experiment, Western blot showed that compared to DMSO control, the downregulation of γ-catenin levels at 7 h pi was significantly rescued (Figure 1E), indicating that γ-catenin reduction is a consequence of proteasome-mediated degradation. To further determine if this degradation occurred through poly-ubiquitination before being processed in the 26S proteasome, we conducted immunoprecipitation using a primary antibody against γ-catenin and then detected poly-ubiquitinated γ-catenin using another antibody against K48-linked poly-ubiquitin. We found that CVB3 infection induced γ-catenin poly-ubiquitination compared to the sham-infected control, while the input γ-catenin level significantly decreased (Figure 1F).

### CVB3-Induced Decrease of Desmosomal Cadherins Leads to Destabilization of γ-Catenin

**FIGURE 2:**
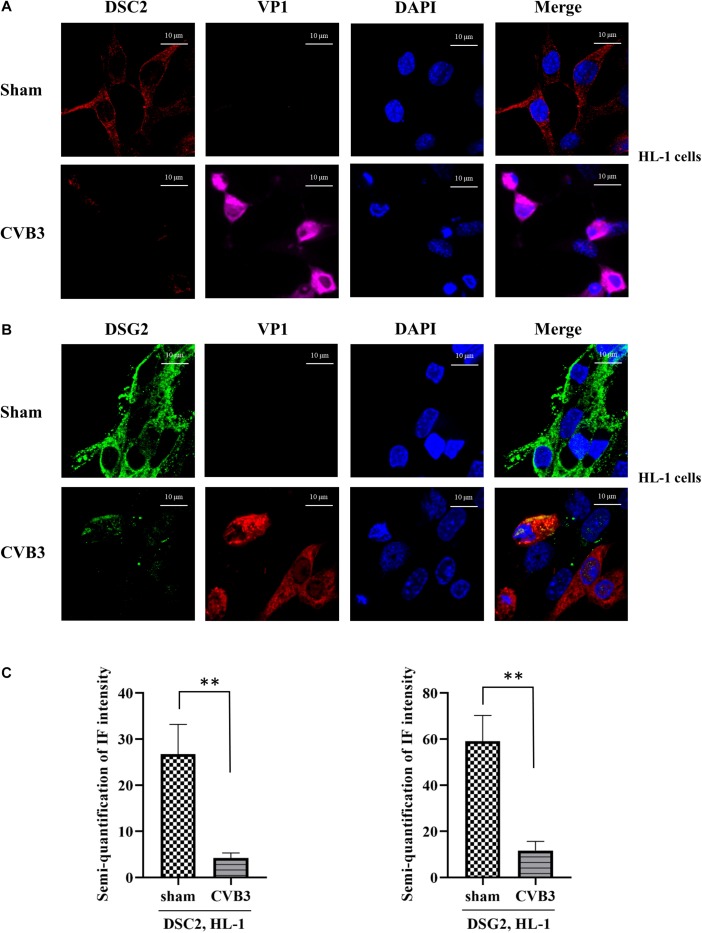
CVB3 induces destabilization of desmosomal cadherins in HL-1 mouse cardiomyocytes. **(A,B)** HL-1 cells were infected with CVB3 at a MOI of 50 for 24 h. Cells were subjected to immunofluorescence staining of DSC-2 (red) **(A)** and DSG-2 (green) **(B)**. Nuclei were stained using DAPI (blue). Images were captured by Zeiss confocal microscope and Zeiss Black program, scale bars = 10 μM. Fluorescence signal intensities were quantified by using ImageJ **(C)**. Data were statistically analyzed, *n* = 3, ^∗∗^*p* < 0.01.

Since the half-life of γ-catenin is known to be prolonged in the desmosome compared to that in the cytoplasm (Kowalczyk et al., 1994), we speculated that CVB3 infection might cause release of γ-catenin from the desmosome into the cytoplasm by destroying its co-localized binding partners in the desmosome, leading to the promotion of γ-catenin degradation. To verify this, we first determined the protein levels and localization of DSC-2 and DSG-2 in CVB3-infected murine HL-1 cardiomyocytes by immunostaining and confocal microscopy. We found that in sham-infected control groups, both DSC-2 and DSG-2 were mainly localized along the cell border where the cells contact each other. After CVB3 infection, however, these protein levels were dramatically decreased and their regular localization pattern was disrupted (Figures 2A,B). Furthermore, the fluorescence signal intensities were also quantified (Figure 2C).

**FIGURE 3:**
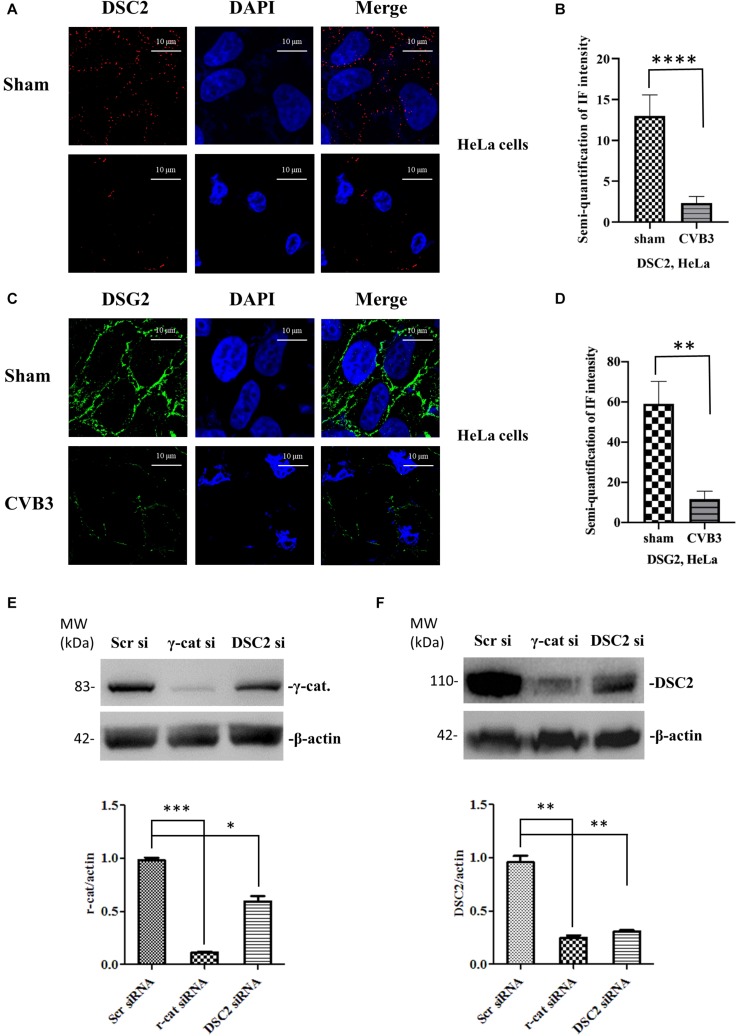
CVB3-induced decrease of desmosomal cadherins leads to reduction of γ-catenin. HeLa cells were infected with CVB3 at a MOI of 10 for 6 h. Cells were then subjected to immunofluorescence staining of DSC-2 (red) **(A,B)** and DSG-2 (green) **(C,D)**, scale bars = 10 μM. Fluorescence signal intensities were quantified as described in Figure 2. HeLa cells were transfected with the indicated specific siRNAs for 48 h and the cell lysates were collected for Western blot analysis of γ-catenin **(E)** and DSC-2 **(F)**. β-actin was used as a loading control. The intensities of the bands were measured using the NIH ImageJ program and normalized to the loading control. Data were statistically analyzed, *n* = 3, ^∗^*p* < 0.05, ^∗∗^*p* < 0.01, ^∗∗∗^*p* < 0.001.

These findings were further verified by immunostaining using CVB3-infected HeLa cells (Figures 3A–D). In addition, to determine whether the silencing of desmosomal cadherins could lead to γ-catenin reduction in the absence of CVB3 infection, HeLa cells were transfected with DSC-2 siRNA or scrambled siRNA, and cell lysates were then collected for Western blot; in parallel, γ-catenin siRNA was used to transfect HeLa cells as a positive control. As shown in Figure 3E, transfection of DSC-2 siRNA led to a ∼45% decrease in γ-catenin protein compared to cells transfected with scrambled siRNAs. Additionally, we found that silencing of γ-catenin could also reduce DSC-2 levels by ∼75% (Figure 3F); this reduction was even higher than that in DSC-2 siRNA-treated cells (∼46%), indicating that γ-catenin and DSC-2 stabilize each other in the desmosome complex.

### DSC-2 Protein Is Cleaved During CVB3 Infection

**FIGURE 4:**
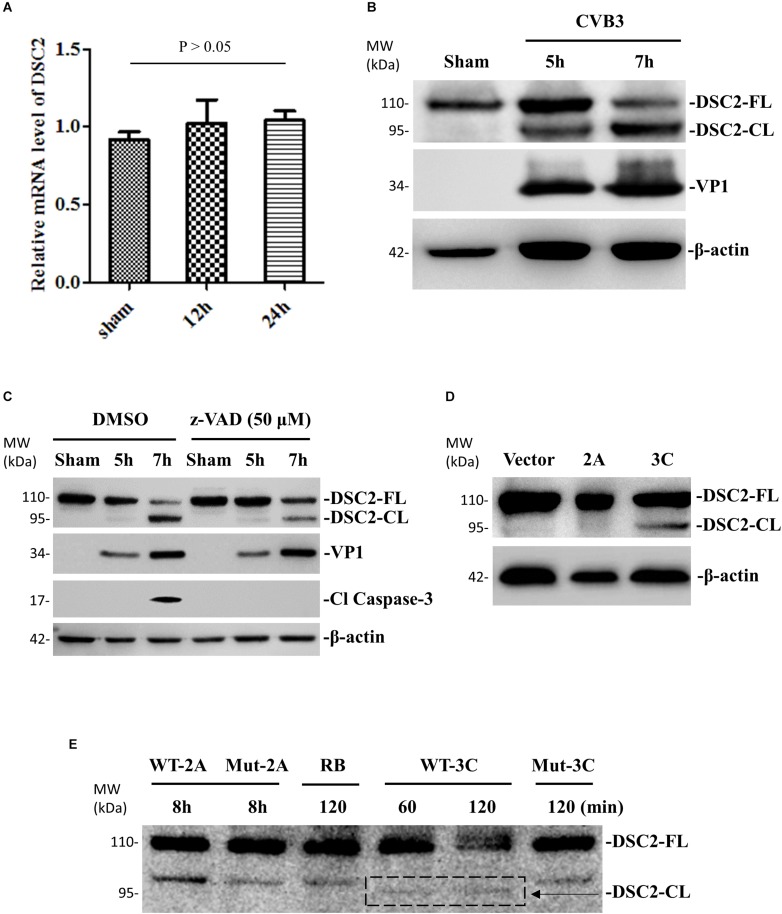
DSC-2 is cleaved during CVB3 infection. **(A)** HeLa cells were infected with CVB3 at a MOI of 10 or sham-infected with PBS and collected at the indicated time points pi. Cellular RNAs were extracted for measuring the mRNA level of DSC-2 by qPCR. Three biological repeats were performed for each assay. Data were normalized to the level of GAPDH in the sample. *p* > 0.05 (not significant). **(B)** HeLa cells were infected or sham-infected as described in **(A)**. Cell lysates were prepared for Western blot analysis of DSC-2 using an anti-N-terminal antibody. VP1 detection was a marker for CVB3 replication. β-actin was used as the loading control. **(C)** HeLa cells were treated with 50 μM z-VAD-fmk or DMSO (negative control), and then infected with CVB3 at a MOI of 10. At the indicated time points pi, the cell lysates were harvested for Western blot analysis using the indicated antibodies. The level of cleaved caspase-3 was used as an indicator of caspase activation. **(D)** HeLa cells were transfected with a viral protease 2A or 3C plasmid, or an empty vector as the negative control. Cell lysates were harvested at 36 h post transfection for Western blot analysis of DSC-2 cleavage products. **(E)** *In vitro* cleavage assay. Non-infected HeLa cell lysates were incubated with recombinant CVB3 wild-type (WT) and mutant (Mut) 2A or 3C protease. At the indicated time points post incubation, the reaction samples were subjected to Western blot analysis of DSC-2 cleavage products. Reaction buffer (RB) only was used as another negative control.

To investigate the effect of CVB3 infection on the expression and integrity of desmosomal cadherins, we first selected DSC-2 as a target and performed qPCR and Western blot to determine DSC-2 mRNA and protein levels after CVB3 infection. We found that CVB3 infection did not alter the mRNA level of DSC-2 (Figure 4A) while Western blot using an anti-N terminal antibody showed the production of a ∼95 kDa band (Figure 4B). Since CVB3 can induce apoptosis by activating caspase-3, we used the pan-caspase inhibitor to test whether the ∼95 kDa fragment was produced by caspase-3 cleavage. We showed that z-VAD-fmk completely blocked caspase-3 cleavage; production of the ∼95 kDa product, however, was only partially inhibited (Figure 4C), suggesting that DSC-2 is cleaved by both CVB3 protease and activated caspase. To identify the viral protease responsible for this cleavage, we transfected HeLa cells with protease expressing plasmids pIRES-2A or pIRES-3C and analyzed the cleavage at 36 h post transfection; results showed that compared to the empty pIRES vector, only the pIRES-3C plasmid induced the production of a ∼95 kDa product (Figure 4D). Meanwhile, we also conducted an *in vitro* cleavage assay using purified CVB3 wild-type and mutant (control) proteases 3C to treat the cell lysates containing DSC-2 protein (Figure 4E). Our data showed that protease 3C, but not 2A, could cleave DSC-2. These data were further supported by a motif search for 3C cleavage site according to a previous publication (Blom et al., 1996). This search found amino acids Q766-G767 as the potential cleavage site (Supplementary Figure S1), which would result in a ∼95 kDa N-terminal fragment, matching the fragment size produced during CVB3 infection.

### DSG-2 Protein Is Cleaved During CVB3 Infection

**FIGURE 5:**
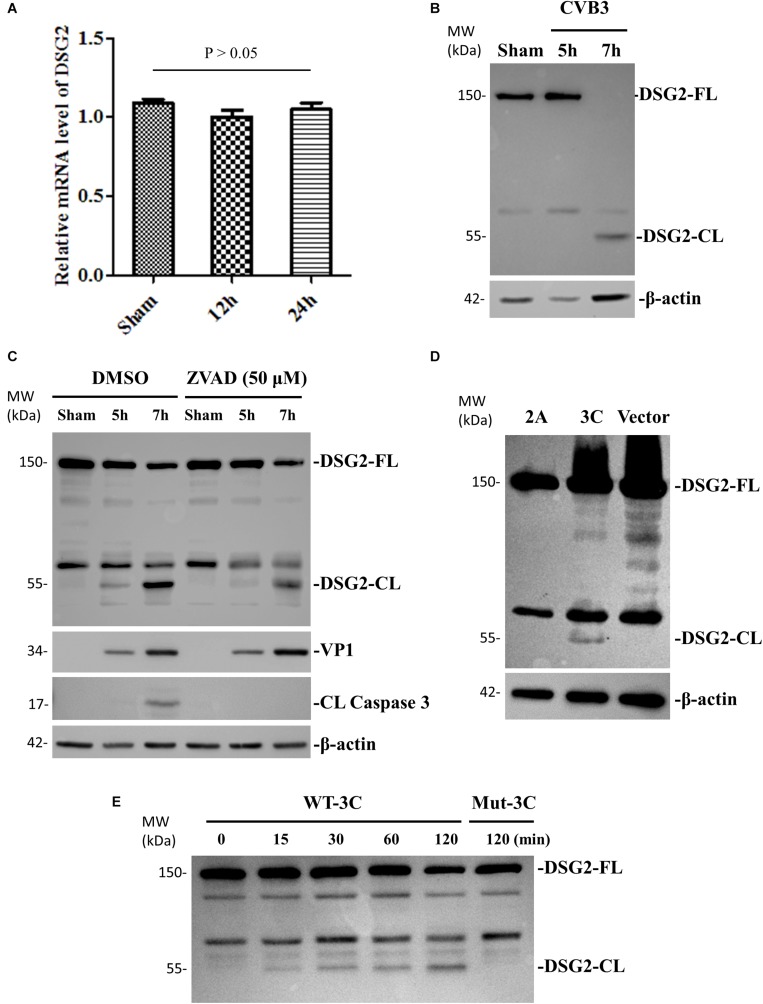
DSG-2 is cleaved during CVB3 infection. **(A)** HeLa cells were infected with CVB3 at a MOI of 10 or sham-infected with PBS and collected at the indicated time points pi. Cellular RNAs were extracted for measuring the mRNA levels of DSG2 by qPCR. Three biological repeats were performed for each assay. Data were normalized to the level of GAPDH in the sample, *p* > 0.05 (not significant). **(B)** HeLa cells were infected or sham-infected as described in **(A)**. Cell lysates were prepared for Western blot analysis of DSG-2 using an anti-DSG-2 antibody targeting the C-terminal of the protein. β-actin level was used as the loading control. **(C)** HeLa cells were treated with 50 μM z-VAD-fmk or DMSO to block caspase activation, and then infected with CVB3 at a MOI of 10. At the indicated time points pi, cellular lysates were subjected to Western blot analysis of DSG-2 cleavage products. **(D)** HeLa cells were transfected with a viral protease 2A or 3C plasmid or an empty vector as the negative control. Cell lysates were harvested at 36 h post transfection for Western blot analysis of DSG-2 cleavage products. **(E)** *In vitro* cleavage assay. Non-infected HeLa cell lysates were incubated for the indicated time points with purified recombinant CVB3 wild-type and mutant 3C protease. Lysates were subjected to Western blot analysis of DSG-2 cleavage products.

Next, we used the same experimental design and methods to verify that DSG-2 gene was not downregulated at the transcriptional level but its protein was cleaved during CVB3 infection, producing a ∼55-kDa C-terminal fragment (Figures 5A,B). We confirmed that both CVB3 protease 3C and cellular caspase could execute this cleavage. However, CVB3 2A protease could not (Figures 5C–E).

### γ-Catenin Suppresses the Activity of Wnt/β-Catenin Signaling

**FIGURE 6:**
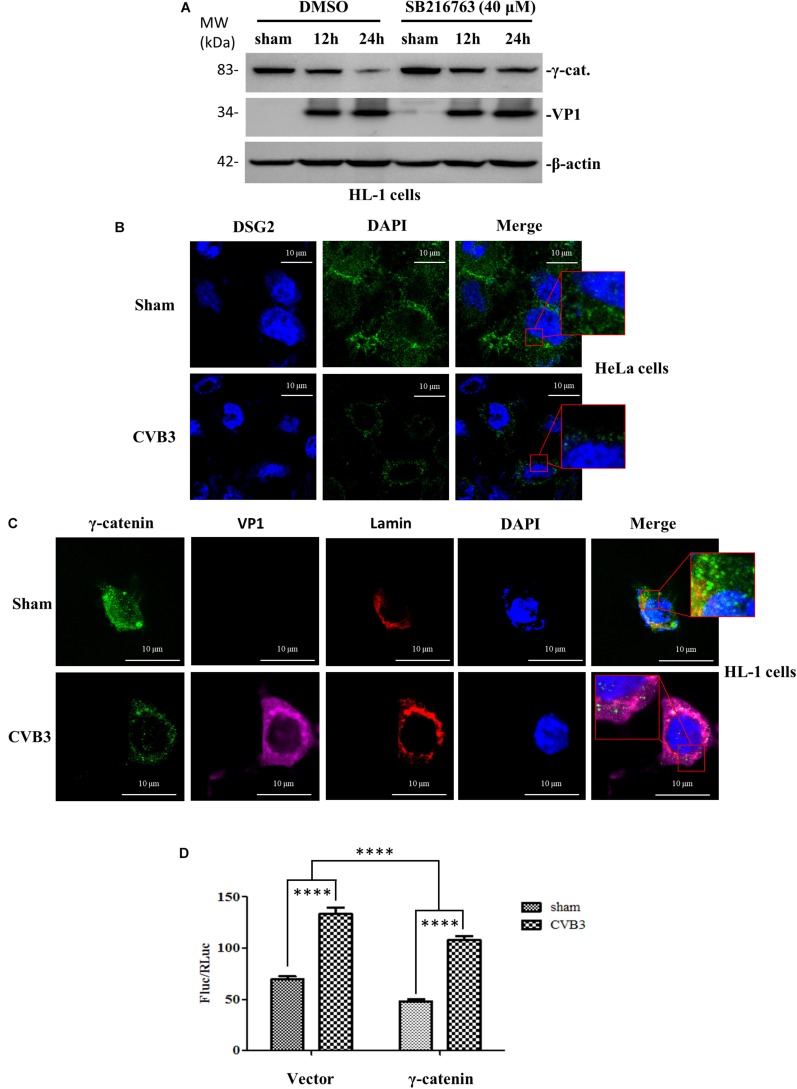
γ-Catenin suppresses the activity of Wnt/β-catenin signaling. **(A)** Murine HL-1 cardiomyocytes were treated with SB216763 or DMSO, and then infected with CVB3 at a MOI of 50 or sham-infected. Cell lysates were collected at the indicated time points pi and subjected to Western blot analysis of γ-catenin. β-actin was used as the loading control. **(B,C)** HeLa cells and HL-1 cardiomyocytes were infected with CVB3 at a MOI of 10 and 50 for 6 h and 24 h, respectively. Cells were subjected to immunofluorescence staining to detect the γ-catenin (green), lamin (red) and CVB3 VP1 protein (purple). Nuclei were stained using DAPI (blue). Images were captured by confocal microscopy, scale bars = 10 μM. The insets show the magnified regions of colocalization. **(D)** HeLa cells were co-transfected with the γ-catenin plasmid or the empty vector and two luciferase reporters (firefly and Renilla luciferase plasmids) for 36 h, and then infected with CVB3 for 6 h. The cell lysates were harvested for dual luciferase assay to detect the relative luciferase activities (Firefly/Renilla). Data were statistically analyzed, *n* = 9, ^****^p < 0.0001.

To determine the role of γ-catenin in Wnt/β-catenin signaling during CVB3 infection, we first focused on glycogen synthase kinase 3 (GSK-3), an important kinase in Wnt signaling regulation that works via phosphorylation and destabilization of β-catenin (Wu and Pan, 2010). We treated HL-1 cardiomyocytes with 40 μM SB216763, an inhibitor of GSK-3, and then infected cells with CVB3. Western blot showed that the reduction of γ-catenin was rescued in SB216763-treated cells compared to DMSO-treated cells (Figure 6A), indicating that γ-catenin is also a target of GSK-3 and may play an important role in Wnt/β-catenin signaling. To determine the localization of γ-catenin during Wnt signal activation, we performed immunostaining using HeLa cells and HL-1 cardiomyocytes and both showed that γ-catenin was translocated to perinucleus upon CVB3 infection (Figures 6B,C). This translocation may enable γ-catenin to participate in the interaction with the LEF/TCF transcription factor and affect the Wnt signaling. To identify the nature of this effect, a luciferase assay was conducted by co-transfection of HeLa cells with two reporters (firefly luciferase and Renilla luciferase) and γ-catenin plasmid (or empty vector) for 36 h. After CVB3 infection, a dual luciferase assay was performed to determine the ratio (FLuc/RLuc) of these two luciferases (Figure 6D). The results showed that γ-catenin overexpression inhibited the activation of Wnt/β-catenin signaling.

### γ-Catenin Inhibits Target Gene Transcription of Wnt/β-Catenin Signaling During CVB3 Infection

**FIGURE 7:**
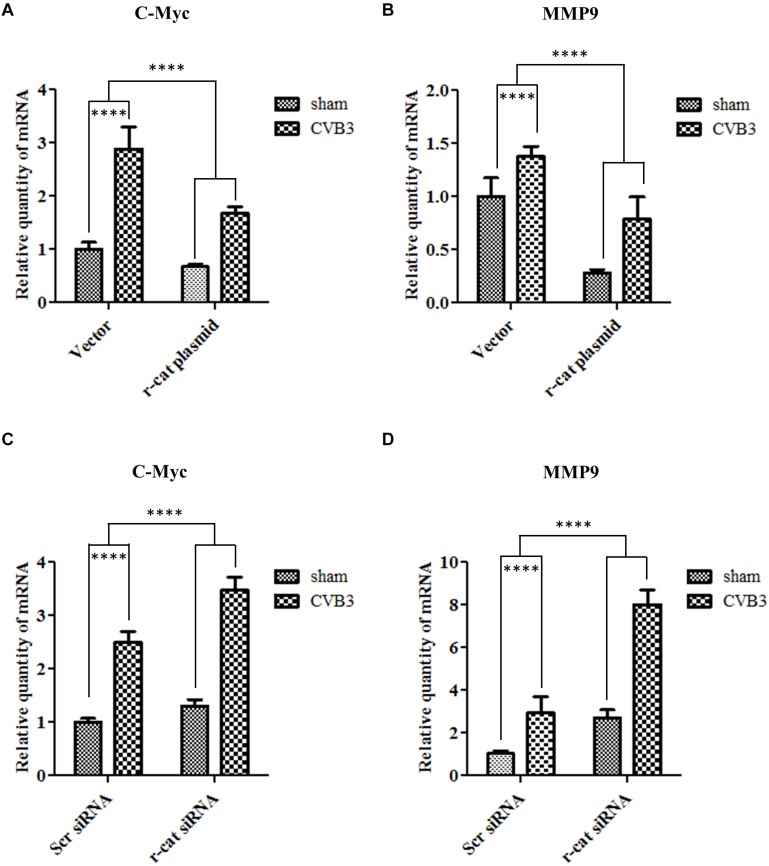
γ-Catenin inhibits target gene transcription of Wnt/β-catenin signaling during CVB3 infection. **(A,B)** HeLa cells were transfected with the γ-catenin plasmid for 36 h to overexpress γ-catenin and then infected with CVB3 at a MOI of 10 or sham-infected. Cellular RNAs were extracted at 6 h pi and then subjected to qPCR analysis of c-myc (A) and MMP9 (B) mRNAs using specific primers. **(C,D)** HeLa cells were transfected with γ-catenin siRNA for 48 h to silence γ-catenin expression and then infected with CVB3 at a MOI of 10 or sham-infected. Cellular RNAs were extracted at 6 h pi and the mRNA levels of c-myc and MMP9 were measured as described in **(A)**. The transcriptional levels of target genes were normalized to that of GAPDH and statistically analyzed, *n* = 3, ^****^p < 0.0001.

Having identified the role of γ-catenin in inhibiting Wnt/β-catenin signaling using a luciferase assay, our next step was to further confirm this notion by detecting the expression of Wnt signaling target genes. To this end, qPCR was conducted to detect transcription of c-myc and MMP9 using total RNAs isolated from HeLa cells transfected with γ-catenin plasmid and infected with CVB3 for 6 h. Results demonstrated that CVB3 infection upregulated the transcription of these two genes, indicating that CVB3 infection induces activation of Wnt signaling. Overexpressing γ-catenin, however, decreased transcription of both c-myc and MMP9 compared to their respective vector-transfected cells (Figures 7A,B). These results suggest that γ-catenin plays an inhibitory role in the Wnt/β-catenin signaling pathway. These data were further solidified by silencing γ-catenin with its specific siRNAs, showing that decreased γ-catenin expression upregulated the transcription of c-myc and MMP9, compared to the transcription in control cells treated with scrambled siRNAs (Figures 7C,D).

### γ-Catenin Expression Inhibits CVB3 Replication While Knockdown of γ-Catenin Expression Enhances CVB3 Replication

**FIGURE 8:**
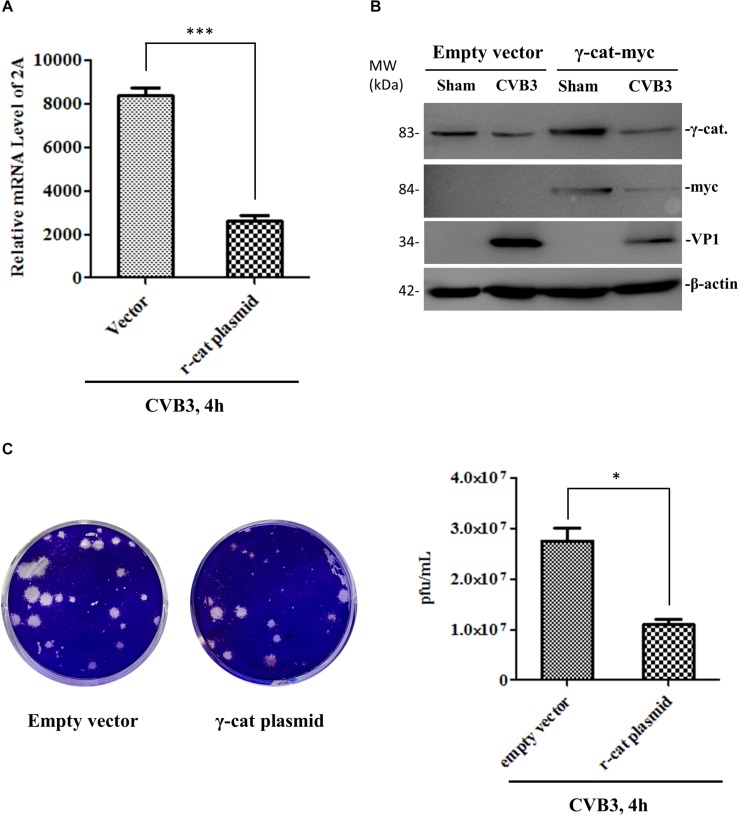
γ-Catenin expression inhibits CVB3 replication. HeLa cells were transfected with the γ-catenin-myc plasmid or empty vector for 36 h and then infected with CVB3 for 4 h. Part of the cell culture was used for cellular RNA extraction to measure the RNA level of CVB3 2A by qPCR **(A)**, and the other part of the culture was used for cell lysate preparation to detect CVB3 VP1 and other proteins by Western blot using the indicated antibodies **(B)**. The cell culture supernatants were used for plaque assay to measure the CVB3 particle release **(C)**. Data were statistically analyzed and graphically presented, *n* = 3, ^∗^*p* < 0.05, ^∗∗∗^*p* < 0.001.

**FIGURE 9:**
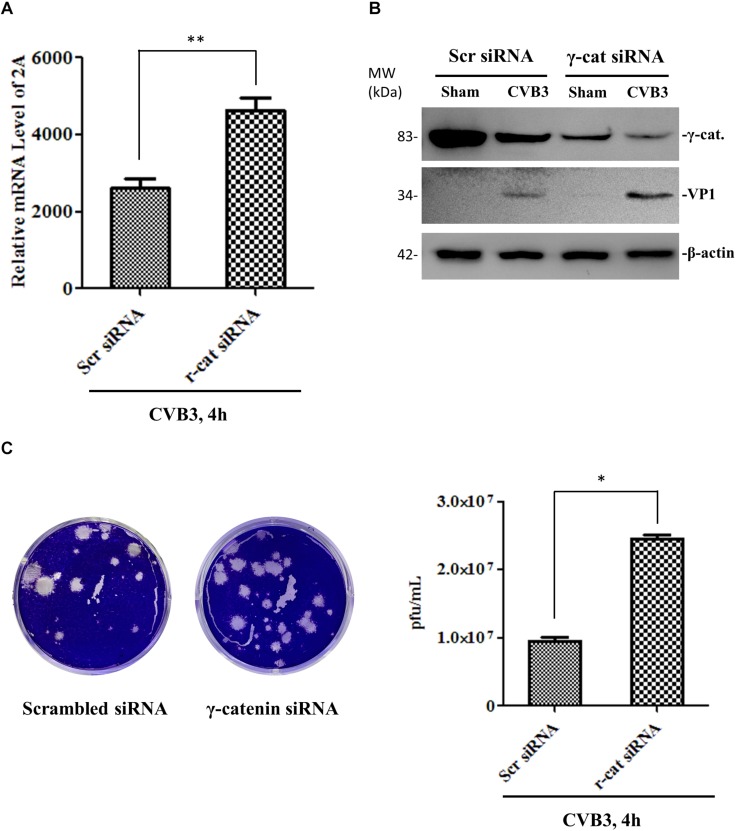
Knockdown of γ-catenin expression enhances CVB3 replication. HeLa cells were transfected with γ-catenin siRNA or scrambled siRNA for 48 h and then infected with CVB3 at a MOI of 10 or sham-infected for 4 h. Part of the cell culture was used for qPCR to measure CVB3 2A RNA **(A),** and the other part was used either for Western blot analysis of CVB3 VP1 protein **(B)** or for plaque assay to determine the released viral particles **(C)**. Data were statistically analyzed and graphically presented, *n* = 3, ^∗^*p* < 0.05, ^∗∗^*p* < 0.01.

To determine the effect of γ-catenin on viral replication, we transfected HeLa cells with a plasmid expressing myc-tagged γ-catenin for 36 h and then infected with CVB3. At 4 h pi, the cell culture was divided into two parts. One part was used to extract total RNAs for qPCR measurement of CVB3 RNA using primers targeting the CVB3 2A gene. The other part was used for (i) Western blotting analysis of CVB3 VP1 using total proteins, and (ii) plaque assay of viral particle formation using culture supernatants. Results showed that γ-catenin overexpression dramatically reduced the levels of 2A RNA (Figure 8A), VP1 protein (Figure 8B) and CVB3 particle release (Figure 8C) compared to the vector-transfected cells, indicating that γ-catenin has a potent inhibitory role in CVB3 replication. These data were further substantiated by siRNA-mediated silencing of γ-catenin and detection of CVB3 RNA, VP1 protein and particle formation using the methods described above. As expected, silencing γ-catenin promoted CVB3 replication, which was demonstrated by the increased levels of CVB3 RNA, VP1 protein, and particle formation (Figures 9A–C).

### γ-Catenin Supports the Induction of Interferon-β and Interferon-Stimulated Genes

**FIGURE 10:**
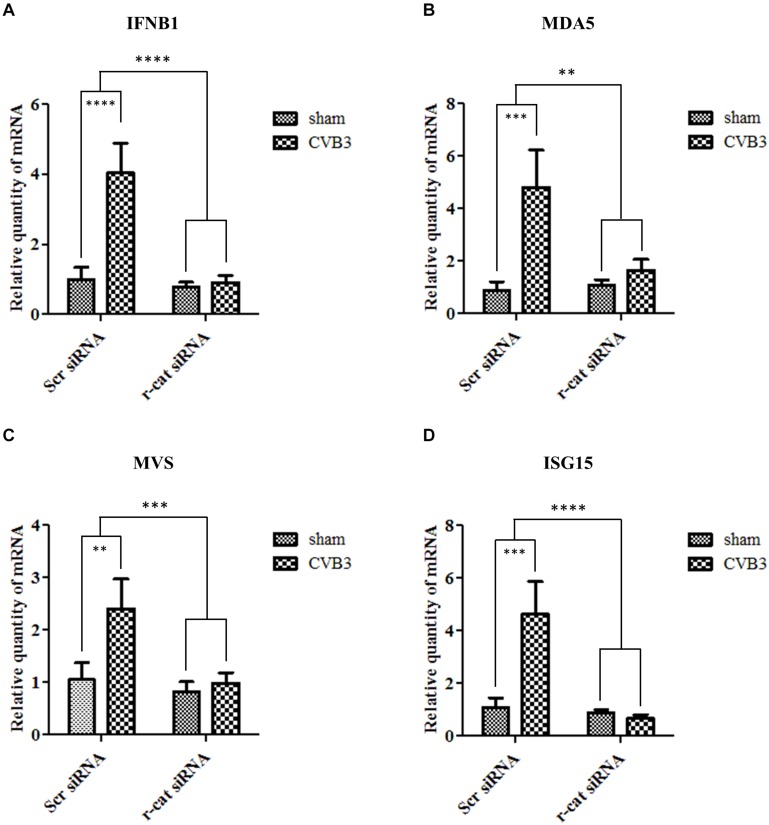
γ-Catenin supports the induction of interferon β1 and its stimulated genes. HeLa cells were transfected with siRNA to knockdown the expression of γ-catenin and then infected with CVB3 at 10 MOI. Scrambled siRNA was used as a negative control. Cellular RNAs were extracted at 6 h pi and then subjected to qPCR analysis of the mRNA levels of IFNB1 gene **(A)**, MDA5 **(B)**, MAVS **(C)**, and ISG15 **(D)**. The transcriptional level of each gene was normalized to GAPDH level and statistically analyzed, *n* = 3, ^∗∗^*p* < 0.01, ^∗∗∗^*p* < 0.001, ^****^p < 0.0001.

To further determine the mechanism by which γ-catenin inhibits CVB3 replication, we selected several mediators in type I interferon (IFN) immune responses for further investigation. We treated cells with γ-catenin siRNA or scrambled siRNA before CVB3 infection and then isolated the cellular RNAs to measure the transcription levels of IFNB1, MDA5 (melanoma differentiation-associated gene 5), MAVS (mitochondrial antiviral-signaling protein) and ISG15 (interferon-stimulated gene of 15 kDa) using qPCR. Data demonstrated that silencing γ-catenin significantly decreased the CVB3-activated transcription of IFNB1 (Figure 10A) and its ISGs (Figures 10B–D) compared to the scrambled siRNA-treated and CVB3-infected cells. These data suggest that γ-catenin expression is essential for CVB3-induced type I IFN immune response.

## Discussion

**FIGURE 11:**
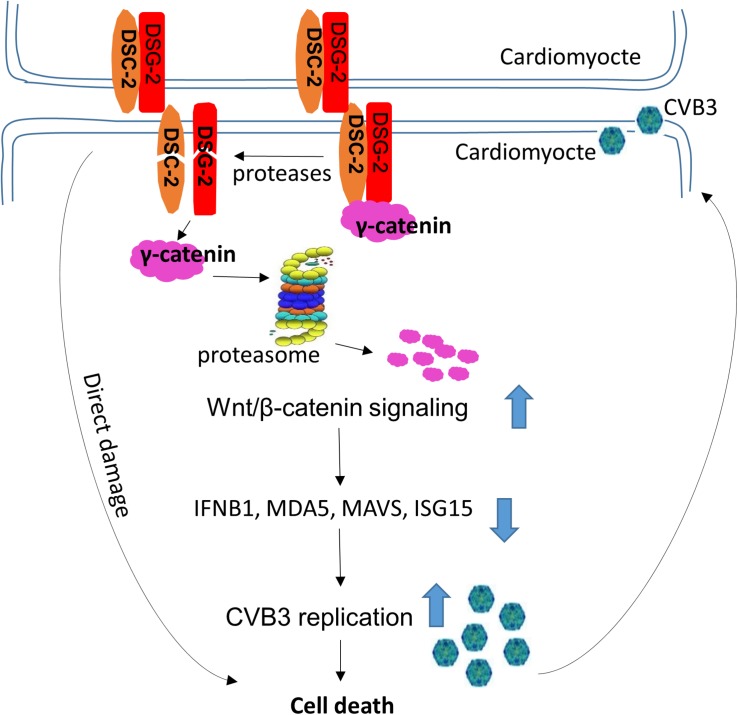
Proposed model of action and mechanism by which CVB3 infection causes cardiomyocyte death. CVB3 infection causes cleavage of desmosomal cadherins by both CVB3 protease and virus-activated caspase, which leads to the direct damage of cardiomyocytes and the release of γ-catenin from the desmosomal complex to the cytosol, where it degrades through the ubiquitin proteasome pathway. The degradation of γ-catenin benefits Wnt/β-catenin signaling and downregulates the IFN-β antiviral immune response, which promotes CVB3 replication and eventually causes cell death. Large arrows indicate up- or down-regulation.

We found that both DSC-2 and DSG-2 were cleaved during CVB3 infection. These cleavages likely cause a domino effect, resulting in the destruction of other partner proteins, such as γ-catenin, in the desmosome complex. We speculated that cleavage of DSC-2 or DSG-2 led to the release of γ-catenin into the cytosol, where it is quickly degraded through the ubiquitin proteasome pathway. This speculation was verified by using proteasome inhibitor MG132. We further found that γ-catenin reduction benefited Wnt/β-catenin signaling and enhanced viral replication by suppressing the interferon-β antiviral immune response. These findings may provide a two-fold explanation of the pathogenesis of CVB3 infection (Figure 11): first, the cleavage of ICD structural proteins may directly damage myocardium architecture, leading to cardiomyocyte death and subsequent heart dysfunction since our previous studies and others have shown that CVB3 infection can cause cardiomyocyte apoptosis (Henke et al., 2000; Jensen et al., 2013); second, reduction of γ-catenin levels in the cell may increase viral load and promote viral pathogenicity.

Previously, it was unknown which protease(s) is/are responsible for the cleavage of DSG-2. Several proteases, such as caspase-8 (Yulis et al., 2018) and ADAM-17 (matrix metalloprotease 17) (Wang et al., 2015), have been shown to be involved in generating a DSG-2 fragment in experiments using human epithelial cells. Caspase-8 cleavage produces a 50-kDa intracellular fragment during cell apoptosis induced by IFN-γ or TNF-α, while cellular ADAM-17 can produce an 80-kDa extracellular fragment of DSG-2 during adenovirus infection. In our study using cardiotropic CVB3 infection, we found that besides virus-activated caspase-3, CVB3 protease 3C, but not 2A, could also cleave DSG-2 and generate a ∼55-kDa intracellular fragment during infection. According to the size of the cleavage products, the caspase and CVB3 3C likely cut the protein at sites that are very close to each other. This would explain why only one single cleavage band (50–55 kDa) was observed on the Western blot membrane. For DSC-2, it was reported that this protein cannot be cleaved by caspases during apoptosis induced by camptothecin or IFN-γ (Nava et al., 2007). However, our experiments showed that both CVB3 protease 3C and the virus-activated caspase-3 could cleave DSC-2, since the pan-caspase inhibitor z-VAD-fmk only partially decreased the amount of DSC-2 cleavage products. It is worth noting that z-VAD-fmk has been reported to have antiviral activities via inhibition of viral proteases (Martin et al., 2007). Thus, the decrease of DSC-2 cleavage fragments during z-VAD-fmk treatment was probably due to either the inhibition of viral protease activity or the blockage of caspase activation, or both, which may explain the inconsistency of our observation with the previous report.

Intercalated disk component proteins not only maintain the architecture of the myocardium, but also participate in signaling transduction pathways (Zhao et al., 2019). Thus, our next goal was to elucidate the effect of CVB3-induced cleavage of desmosome proteins on signal transduction. DSG-2 and DSC-2 are transmembrane anchoring components that stabilize the desmosome structure and serve as bridging cables that connect the intracellular components (Li and Radice, 2010; Sohier et al., 2018). γ-Catenin is a cytoplasmic protein that binds to the intracellular parts of desmosomal cadherins. It has been reported that γ-catenin is rapidly degraded when expressed in a fibroblast model system in the absence of interactions with either desmoglein or desmocollin (Kowalczyk et al., 1994). As a member of the armadillo family of proteins and a paralog of β-catenin, γ-catenin also participates in cell signaling in addition to its role as a structural protein (Manring et al., 2018). The protein level of γ-catenin was dramatically decreased during CVB3 infection and no significant amount of cleaved products was observed in our Western blot analysis. This implies that CVB3-induced downregulation of γ-catenin mainly occurs through post-translational degradation. This speculation was further verified via co-immunoprecipitation assay of the poly-ubiquitinated γ-catenin.

γ-Catenin and β-catenin share many common interacting protein partners, such as cadherins, LEF/TCF transcription factors, and the APC/Axin degradation machinery, and these proteins can fulfill certain common functions (Zhurinsky et al., 2000b; Zhao et al., 2019). Furthermore, the competition between these two catenin proteins mediates the cross-talk of cadherin-based adhesion, catenin-dependent transcription and Wnt signaling (Zhurinsky et al., 2000a). However, how γ-catenin affects Wnt signaling is still debatable. For instance, using a human malignant mesothelioma cell line, researchers found that γ-catenin has TCF/LEF family-dependent transcriptional activity in a β-catenin deficient cell line (Maeda et al., 2004). This implies that γ-catenin may compete with β-catenin for binding to TCF/LEF family members and thus inhibit Wnt signaling. Another group reported that Wnt-3a activation of LEF/TCF-dependent transcription depends on β-catenin, but not on γ-catenin (Shimizu et al., 2008). However, the role of γ-catenin in the Wnt signaling – particularly in the setting of picornavirus infection – has not been studied. Our results showed that degradation of γ-catenin was suppressed when treating the cells with GSK3 inhibitor SB216763, indicating that β-catenin and γ-catenin potentially compete to serve as a phosphorylation target of GSK3 kinase. C-myc and MMP9 are two target genes of Wnt signaling associated with heart development (Foulquier et al., 2018). We found that overexpression of γ-catenin downregulated the transcription of these two genes, implying the suppression of Wnt/β-catenin signaling by γ-catenin. MMP9 is reported to be induced by Wnt signaling during the course of vascular calcifications (Freise et al., 2016), and is also induced during CVB3 infection (Cheung et al., 2008). Our qPCR analysis showed that γ-catenin-induced suppression of Wnt signaling reduced the transcriptional level of MMP9, but that CVB3 infection rescued this reduction. These data are consistent with the above-mentioned reports. In addition, c-myc was also decreased by the overexpression of γ-catenin and recovered by CVB3 infection. In summary, our data support the previous reports that γ-catenin plays an inhibitory role in Wnt/β-catenin signaling.

In searching for the central role of γ-catenin in viral pathogenesis, we found that γ-catenin overexpression reduced CVB3 particle formation, suggesting an antiviral effect for γ-catenin. To reveal the underlying mechanism by which γ-catenin activates an antiviral effect during CVB3 infection, we focused on the type I interferon response. This is because type I IFNs are produced early during viral infection; mice deficient in type I IFN receptor display delayed CVB3 clearance and accelerated myocardial disease, and die more rapidly than wild-type mice (Wessely et al., 2001; Althof et al., 2014). More importantly, β-catenin and γ-catenin have recently been reported to be important regulators in innate immune response, suppressing influenza A virus through induction of expression of IFNB1 and its related genes, such as RIG-1, MDA5, MAVS, etc. (Hillesheim et al., 2014). Similarly, our data demonstrated that γ-catenin significantly enhanced the expression of interferon-β by activating the MDA5/MAVS pathway. MDA5, like RIG-1, is an important mediator of intracellular viral nucleic acid sensing and uses the signaling adaptor (MAVS) to coordinate the activation of interferon regulatory factor-3 to induce the production of the type I IFNs (Kawai et al., 2005; Seth et al., 2005). MDA5 and RIG-I both consist of a C-terminal DEXD/H-box RNA-helicase domain and an N-terminal caspase recruitment domain and can induce IFN gene transcription in response to dsRNA (Yoneyama et al., 2005). CVB3, as a single-stranded RNA virus, replicates its genome via production of a dsRNA intermediate. Thus, MDA5 likely plays a role in the immune response to CVB3 infection, inducing IFN gene transcription upon recognition of the CVB3 dsRNA intermediate. Finally, we also determined the expression of another IFN-stimulated gene, ISG15, a small ubiquitin family protein playing an important antiviral role via ubiquitin-like modification of viral or host proteins (Loeb and Haas, 1992; Albert et al., 2018). ISG15 has been shown to defend against heart failure in virus-induced cardiomyopathy (Rahnefeld et al., 2014). We therefore also measured the expression of ISG15 as the target gene of type I interferon response. As expected, ISG15 was dramatically increased during CVB3 infection; however, when γ-catenin was silenced with siRNAs, the ISG15 expression levels were reduced to their basal level in both CVB3-infected and sham-infected cells. These data, combined with the detection of other ISG gene expression, indicate that γ-catenin expression suppresses CVB3 replication via induction of the type I IFN signaling and activation of antiviral ISGs.

## Conclusion

We have provided the first evidence on how CVB3 destroys the integrity of desmosome by cleaving DSC-2 and DSG-2. The destruction of desmosomal cadherins causes γ-catenin to be released into the cytosol for further degradation via the ubiquitin-proteasome pathway. Since γ-catenin executes its anti-CVB3 function through activation of the IFN-β immune response, virus-induced degradation of γ-catenin benefits CVB3 replication, enhancing viral pathogenicity. Based on this new understanding of the underlying mechanism by which CVB3 infection causes direct damage or death of cardiomyocytes, searching for novel inhibitors to block cleavage and prevent γ-catenin degradation could be an effective strategy for therapeutic intervention to protect the integrity of the myocardium and treat viral myocarditis.

## Data Availability Statement

All datasets generated for this study are included in the article/Supplementary Material.

## Ethics Statement

The animal study was reviewed and approved by the Animal Care Committee, Faculty of Medicine, University of British Columbia. Animal work was conducted by strictly following the Guide to the Care and Use of Experimental Animals – Canadian Council on Animal Care.

## Author Contributions

DY, XY, and GZ conceived the study. GZ, HZ, and YQ conducted the experiments and data analyses. GZ and DY wrote the manuscript. All authors read and approved the final manuscript.

## Conflict of Interest

The authors declare that the research was conducted in the absence of any commercial or financial relationships that could be construed as a potential conflict of interest.

## References

[B1] Albert M. Becares M. Falqui M. Fernandez-Lozano C. Guerra S. (2018). ISG15, a Small Molecule with Huge Implications: regulation of Mitochondrial Homeostasis. *Viruses* 10:E629. 10.3390/v10110629 PMC6265978 30428561

[B2] Althof N. Harkins S. Kemball C. C. Flynn C. T. Alirezaei M. Whitton J. L. (2014). In vivo ablation of type I interferon receptor from cardiomyocytes delays coxsackieviral clearance and accelerates myocardial disease. *J. Virol.* 88 5087–5099. 24574394 10.1128/JVI.00184-14 PMC3993796

[B3] Blom N. Hansen J. Blaas D. Brunak S. (1996). Cleavage site analysis in picornaviral polyproteins: discovering cellular targets by neural networks. *Protein Sci.* 5 2203–2216. 8931139 10.1002/pro.5560051107 PMC2143287

[B4] Campuzano O. Alcalde M. Allegue C. Iglesias A. Garcia-Pavia P. Partemi S. (2013). Genetics of arrhythmogenic right ventricular cardiomyopathy. *J. Med. Genet.* 50 280–289. 23468208 10.1136/jmedgenet-2013-101523

[B5] Carvajal-Huerta L. (1998). Epidermolytic palmoplantar keratoderma with woolly hair and dilated cardiomyopathy. *J. Am. Acad. Dermatol.* 39 418–421. 9738775 10.1016/s0190-9622(98)70317-2

[B6] Cheung C. Marchant D. Walker E. K. Luo Z. Zhang J. Yanagawa B. (2008). Ablation of matrix metalloproteinase-9 increases severity of viral myocarditis in mice. *Circulation* 117 1574–1582. 18332263 10.1161/CIRCULATIONAHA.107.733238

[B7] Davies M. J. McKenna W. J. (1994). Dilated cardiomyopathy: an introduction to pathology and pathogenesis. *Br. Heart J.* 72 S24. 10.1136/hrt.72.6_suppl.s24 PMC1025672 7873320

[B8] Fairweather D. Stafford K. A. Sung Y. K. (2012). Update on coxsackievirus B3 myocarditis. *Curr. Opin. Rheumatol.* 24 401–407. 22488075 10.1097/BOR.0b013e328353372d PMC4536812

[B9] Foulquier S. Daskalopoulos E. P. Lluri G. Hermans K. C. M. Deb A. Blankesteijn W. M. (2018). WNT signaling in cardiac and vascular disease. *Pharmacol. Rev.* 70 68–141. 29247129 10.1124/pr.117.013896 PMC6040091

[B10] Freise C. Kretzschmar N. Querfeld U. (2016). Wnt signaling contributes to vascular calcification by induction of matrix metalloproteinases. *BMC Cardiovasc. Disord.* 16:185. 10.1186/s12872-016-0362-8 27716072 PMC5045611

[B11] Henke A. Launhardt H. Klement K. Stelzner A. Zell R. Munder T. (2000). Apoptosis in coxsackievirus B3-caused diseases: interaction between the capsid protein VP2 and the proapoptotic protein siva. *J. Virol.* 74 4284–4290. 10756043 10.1128/jvi.74.9.4284-4290.2000 PMC111945

[B12] Hillesheim A. Nordhoff C. Boergeling Y. Ludwig S. Wixler V. (2014). beta-catenin promotes the type I IFN synthesis and the IFN-dependent signaling response but is suppressed by influenza A virus-induced RIG-I/NF-kappaB signaling. *Cell Commun. Signal.* 12 29. 10.1186/1478-811X-12-29 PMC4021428 24767605

[B13] Jagdeo J. M. Dufour A. Klein T. Solis N. Kleifeld O. Kizhakkedathu J. (2018). N-terminomics TAILS identifies host cell substrates of poliovirus and coxsackievirus B3 3C proteinases that modulate virus infection. *J. Virol.* 92:e02211-17. 10.1128/JVI.02211-17 PMC5874412 29437971

[B14] Jensen K. J. Garmaroudi F. S. Zhang J. Lin J. Boroomand S. Zhang M. (2013). An ERK-p38 subnetwork coordinates host cell apoptosis and necrosis during coxsackievirus B3 infection. *Cell Host Microbe.* 13 67–76. 23332156 10.1016/j.chom.2012.11.009 PMC3553504

[B15] Kawai T. Takahashi K. Sato S. Coban C. Kumar H. Kato H. (2005). IPS-1, an adaptor triggering RIG-I- and Mda5-mediated type I interferon induction. *Nat. Immunol.* 6 981–988. 16127453 10.1038/ni1243

[B16] Kowalczyk A. P. Hatzfeld M. Bornslaeger E. A. Kopp D. S. Borgwardt J. E. Corcoran C. M. (1999). The head domain of plakophilin-1 binds to desmoplakin and enhances its recruitment to desmosomes. Implications for cutaneous disease. *J. Biol. Chem.* 274 18145–18148. 10373410 10.1074/jbc.274.26.18145

[B17] Kowalczyk A. P. Palka H. L. Luu H. H. Nilles L. A. Anderson J. E. Wheelock M. J. (1994). Posttranslational regulation of plakoglobin expression. Influence of the desmosomal cadherins on plakoglobin metabolic stability. *J. Biol. Chem.* 269 31214–31223. 7983064

[B18] Laitinen O. H. Svedin E. Kapell S. Nurminen A. Hytonen V. P. Flodstrom-Tullberg M. (2016). Enteroviral proteases: structure, host interactions and pathogenicity. *Rev. Med. Virol.* 26 251–267. 27145174 10.1002/rmv.1883 PMC7169145

[B19] Li J. Radice G. L. (2010). A new perspective on intercalated disc organization: implications for heart disease. *Dermatol. Res. Pract.* 2010:207835. 10.1155/2010/207835 PMC2879923 20585598

[B20] Loeb K. R. Haas A. L. (1992). The interferon-inducible 15-kDa ubiquitin homolog conjugates to intracellular proteins. *J. Biol. Chem.* 267 7806–7813. 1373138

[B21] Lombardi R. da Graca Cabreira-Hansen M. Bell A. Fromm R. R. Willerson J. T. Marian A. J. (2011). Nuclear plakoglobin is essential for differentiation of cardiac progenitor cells to adipocytes in arrhythmogenic right ventricular cardiomyopathy. *Circ. Res.* 109 1342–1353. 22021931 10.1161/CIRCRESAHA.111.255075 PMC3237769

[B22] Maeda O. Usami N. Kondo M. Takahashi M. Goto H. Shimokata K. (2004). Plakoglobin (gamma-catenin) has TCF/LEF family-dependent transcriptional activity in beta-catenin-deficient cell line. *Oncogene* 23 964–972. 14661054 10.1038/sj.onc.1207254

[B23] Manring H. R. Dorn L. E. Ex-Willey A. Accornero F. Ackermann M. A. (2018). At the heart of inter- and intracellular signaling: the intercalated disc. *Biophys. Rev.* 10 961–971. 29876873 10.1007/s12551-018-0430-7 PMC6082301

[B24] Martin U. Jarasch N. Nestler M. Rassmann A. Munder T. Seitz S. (2007). Antiviral effects of pan-caspase inhibitors on the replication of coxsackievirus B3. *Apoptosis* 12 525–533. 17211569 10.1007/s10495-006-0015-y

[B25] Mason J. W. (2003). Myocarditis and dilated cardiomyopathy: an inflammatory link. *Cardiovasc. Res.* 60 5–10. 14522402 10.1016/s0008-6363(03)00437-1

[B26] McManus B. M. Chow L. H. Wilson J. E. Anderson D. R. Gulizia J. M. Gauntt C. J. (1993). Direct myocardial injury by enterovirus: a central role in the evolution of murine myocarditis. *Clin. Immunol. Immunopathol.* 68 159–169. 7689428 10.1006/clin.1993.1113

[B27] Miravet S. Piedra J. Miro F. Itarte E. Garcia, de Herreros A. (2002). The transcriptional factor Tcf-4 contains different binding sites for beta-catenin and plakoglobin. *J. Biol. Chem.* 277 1884–1891. 11711551 10.1074/jbc.M110248200

[B28] Nava P. Laukoetter M. G. Hopkins A. M. Laur O. Gerner-Smidt K. Green K. J. (2007). Desmoglein-2: a novel regulator of apoptosis in the intestinal epithelium. *Mol. Biol. Cell* 18 4565–4578. 17804817 10.1091/mbc.E07-05-0426 PMC2043542

[B29] Qiu Y. Ye X. Zhang H. M. Hanson P. Zhao G. Tong L. (2017). Cleavage of osmosensitive transcriptional factor NFAT5 by coxsackieviral protease 2A promotes viral replication. *PLoS Pathog.* 13:e1006744. 10.1371/journal.ppat.1006744 29220410 PMC5738146

[B30] Rahnefeld A. Klingel K. Schuermann A. Diny N. L. Althof N. Lindner A. (2014). Ubiquitin-like protein ISG15 (interferon-stimulated gene of 15 kDa) in host defense against heart failure in a mouse model of virus-induced cardiomyopathy. *Circulation* 130 1589–1600. 25165091 10.1161/CIRCULATIONAHA.114.009847

[B31] Rao B. H. Reddy I. S. Chandra K. S. (1996). Familial occurrence of a rare combination of dilated cardiomyopathy with palmoplantar keratoderma and curly hair. *Indian Heart J.* 48 161–162. 8682558

[B32] Rasmussen T. B. Nissen P. H. Palmfeldt J. Gehmlich K. Dalager S. Jensen U. B. (2014). Truncating plakophilin-2 mutations in arrhythmogenic cardiomyopathy are associated with protein haploinsufficiency in both myocardium and epidermis. *Circ. Cardiovasc. Genet.* 7 230–240. 24704780 10.1161/CIRCGENETICS.113.000338

[B33] Seth R. B. Sun L. Ea C. K. Chen Z. J. (2005). Identification and characterization of MAVS, a mitochondrial antiviral signaling protein that activates NF-kappaB and IRF 3. *Cell* 122 669–682. 16125763 10.1016/j.cell.2005.08.012

[B34] Sheikh F. Ross R. S. Chen J. (2009). Cell-cell connection to cardiac disease. *Trends Cardiovasc. Med.* 19 182–190. 20211433 10.1016/j.tcm.2009.12.001 PMC3601820

[B35] Shimizu M. Fukunaga Y. Ikenouchi J. Nagafuchi A. (2008). Defining the roles of beta-catenin and plakoglobin in LEF/T-cell factor-dependent transcription using beta-catenin/plakoglobin-null F9 cells. *Mol. Cell. Biol.* 28 825–835. 17984222 10.1128/MCB.02375-06 PMC2223424

[B36] Skern T. Liebig H. D. (1994). Picornains 2A and 3C. *Methods Enzymol.* 244 583–595. 7845234 10.1016/0076-6879(94)44042-5

[B37] Sohier P. Legrand L. Aktary Z. Grill C. Delmas V. Bernex F. (2018). A histopathological classification system of Tyr::NRAS(Q61K) murine melanocytic lesions: a reproducible simplified classification. *Pigment Cell Melanoma Res.* 31 423–431. 29224244 10.1111/pcmr.12677

[B38] Vermij S. H. Abriel H. van Veen T. A. (2017). Refining the molecular organization of the cardiac intercalated disc. *Cardiovasc. Res.* 113 259–275. 28069669 10.1093/cvr/cvw259

[B39] Wang H. Ducournau C. Saydaminova K. Richter M. Yumul R. Ho M. (2015). Intracellular signaling and desmoglein 2 shedding triggered by human adenoviruses Ad3, Ad14, and Ad14P1. *J. Virol.* 89 10841–10859. 26292319 10.1128/JVI.01425-15 PMC4621136

[B40] Wessely R. Klingel K. Knowlton K. U. Kandolf R. (2001). Cardioselective infection with coxsackievirus B3 requires intact type I interferon signaling: implications for mortality and early viral replication. *Circulation* 103 756–761. 11156890 10.1161/01.cir.103.5.756

[B41] Wu D. Pan W. (2010). GSK3: a multifaceted kinase in Wnt signaling. *Trends Biochem. Sci.* 35 161–168. 19884009 10.1016/j.tibs.2009.10.002 PMC2834833

[B42] Yang D. Cheung P. Sun Y. Yuan J. Zhang H. Carthy C. M. (2003). A shine-dalgarno-like sequence mediates in vitro ribosomal internal entry and subsequent scanning for translation initiation of coxsackievirus B3 RNA. *Virology* 305 31–43. 12504538 10.1006/viro.2002.1770

[B43] Ye X. Zhang H. M. Qiu Y. Hanson P. J. Hemida M. G. Wei W. (2014). Coxsackievirus-induced miR-21 disrupts cardiomyocyte interactions via the downregulation of intercalated disk components. *PLoS Pathog.* 10:e1004070. 10.1371/journal.ppat.1004070 24722419 PMC3983067

[B44] Yoneyama M. Kikuchi M. Matsumoto K. Imaizumi T. Miyagishi M. Taira K. (2005). Shared and unique functions of the DExD/H-box helicases RIG-I, MDA5, and LGP2 in antiviral innate immunity. *J. Immunol.* 175 2851–2858. 16116171 10.4049/jimmunol.175.5.2851

[B45] Yuan J. Cheung P. K. Zhang H. M. Chau D. Yang D. (2005a). Inhibition of coxsackievirus B3 replication by small interfering RNAs requires perfect sequence match in the central region of the viral positive strand. *J. Virol.* 79 2151–2159. 15681418 10.1128/JVI.79.4.2151-2159.2005 PMC546545

[B46] Yuan J. Zhang J. Wong B. W. Si X. Wong J. Yang D. (2005b). Inhibition of glycogen synthase kinase 3beta suppresses coxsackievirus-induced cytopathic effect and apoptosis via stabilization of beta-catenin. *Cell Death Differ.* 12 1097–1106. 15905881 10.1038/sj.cdd.4401652

[B47] Yulis M. Quiros M. Hilgarth R. Parkos C. A. Nusrat A. (2018). Intracellular Desmoglein-2 cleavage sensitizes epithelial cells to apoptosis in response to pro-inflammatory cytokines. *Cell Death Dis.* 9:389. 10.1038/s41419-018-0380-9 PMC5844960 29523777

[B48] Zhang H. M. Qiu Y. Zhao G. Wang H. Chen Y. T. Aghakeshmiri S. (2020). Cleavage and degradation of EDEM1 promotes coxsackievirus B3 replication via ATF6a-mediated unfolded protein response signalling. *Cell Microbiol.* e13198. 10.1111/cmi.13198 32083795

[B49] Zhao G. Qiu Y. Zhang H. M. Yang D. (2019). Intercalated discs: cellular adhesion and signaling in heart health and diseases. *Heart Fail Rev.* 24 115–132. 30288656 10.1007/s10741-018-9743-7

[B50] Zhurinsky J. Shtutman M. Ben-Ze’ev A. (2000a). Differential mechanisms of LEF/TCF family-dependent transcriptional activation by beta-catenin and plakoglobin. *Mol. Cell. Biol.* 20 4238–4252. 10825188 10.1128/mcb.20.12.4238-4252.2000 PMC85792

[B51] Zhurinsky J. Shtutman M. Ben-Ze’ev A. (2000b). Plakoglobin and beta-catenin: protein interactions, regulation and biological roles. *J. Cell Sci.* 113(Pt 18), 3127–3139. 10954412 10.1242/jcs.113.18.3127

