# Supplementary figures and images for: Cleavage of Desmosomal Cadherins Promotes γ-Catenin Degradation and Benefits Wnt Signaling in Coxsackievirus B3-Induced Destruction of Cardiomyocytes
Source: Front Microbiol. 2020 May 8;11:767. doi: 10.3389/fmicb.2020.00767 (PMC7225294; doi:10.3389/fmicb.2020.00767)

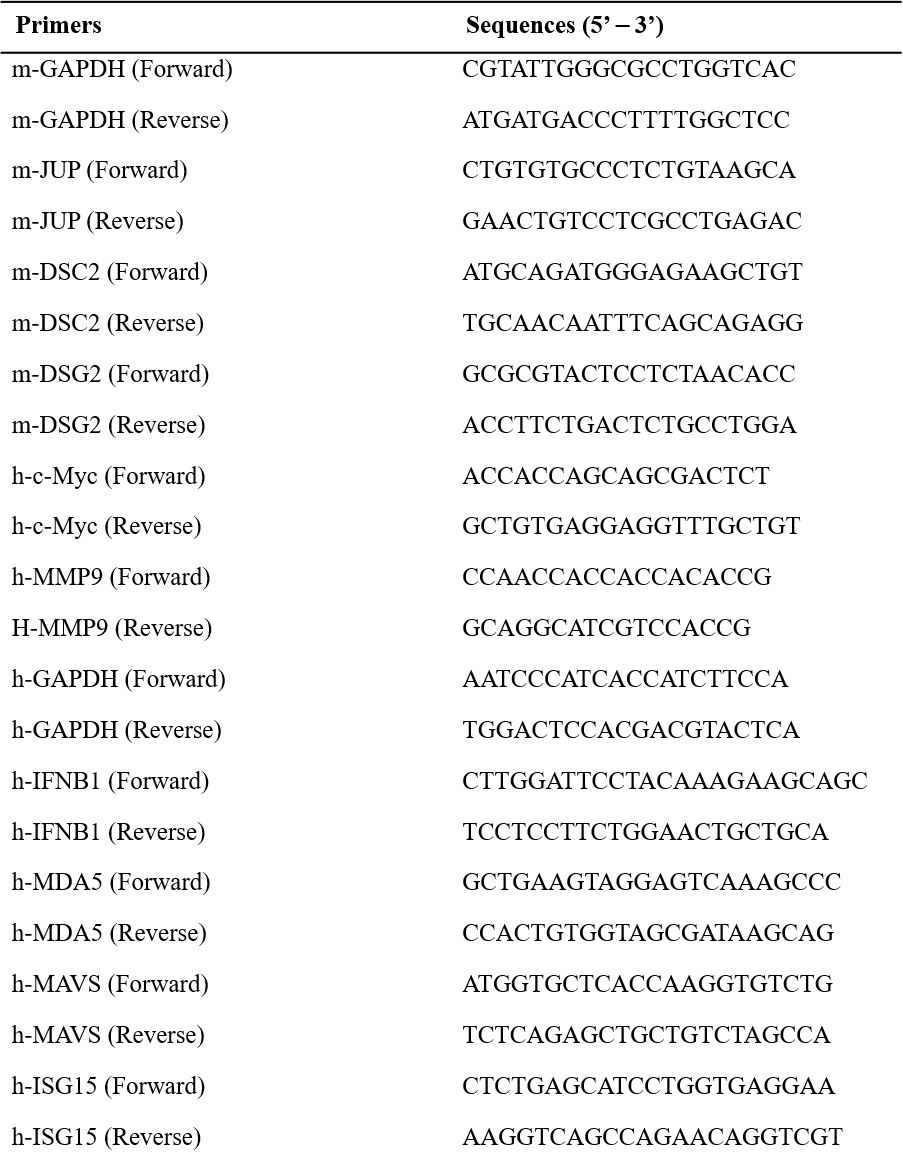


**Supplementary Table 1. qPCR primers used in this study***

* JUP: coding gene for γ-catenin

Supplement: Supplementary file 1 [file Table_1.docx]

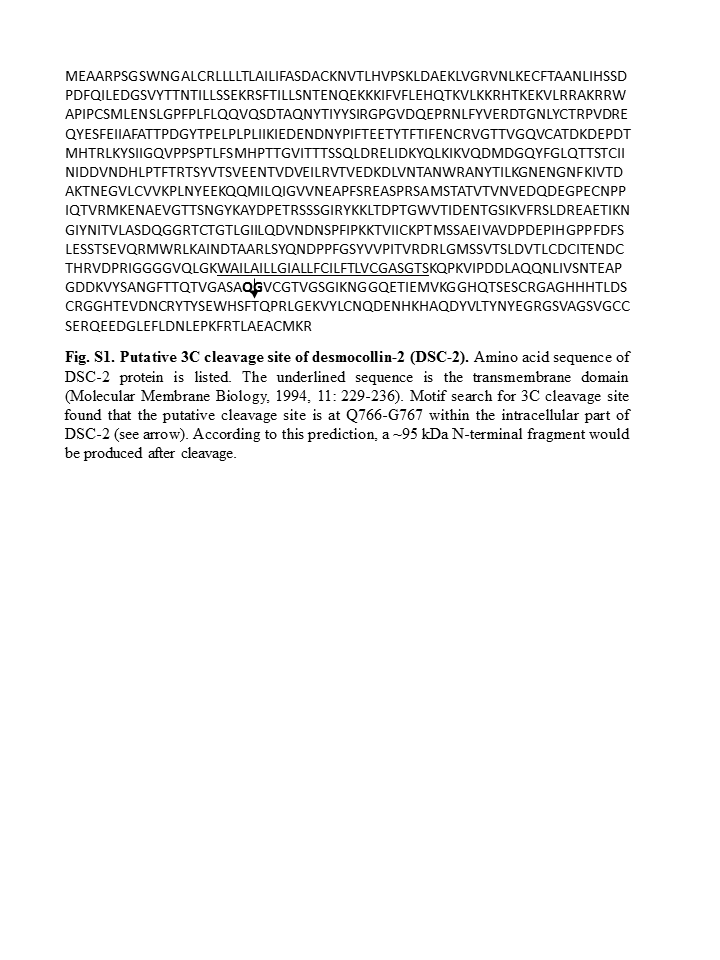

Supplement: Supplementary file 2 [file Image_1.tif]
